# A deep learning model for detection of leukocytes under various interference factors

**DOI:** 10.1038/s41598-023-29331-3

**Published:** 2023-02-07

**Authors:** Meiyu Li, Cong Lin, Peng Ge, Lei Li, Shuang Song, Hanshan Zhang, Lu Lu, Xiaoxiang Liu, Fang Zheng, Shijie Zhang, Xuguo Sun

**Affiliations:** 1 Tianjin Cancer Hospital Airport Hospital, National Clinical Research Center for Cancer, Tianjin, China; 2 School of Intelligent Systems Science and Engineering, Jinan University, Zhuhai, China; 3 Clinical Laboratory, Tianjin Chest Hospital, Tianjin, China; 4 The Australian National University, Canberra, Australia; 5 Institute of Disaster Medicine, Tianjin University, Tianjin, China; 6 School of Medical Laboratory, Tianjin Medical University, Tianjin, China; 7 Department of Pharmacology, School of Basic Medical Sciences, Tianjin Medical University, Tianjin, China

**Keywords:** Medical research, Mathematics and computing

## Abstract

The accurate detection of leukocytes is the basis for the diagnosis of blood system diseases. However, diagnosing leukocyte disorders by doctors is time-consuming and requires extensive experience. Automated detection methods with high accuracy can improve detection efficiency and provide recommendations to inexperienced doctors. Current methods and instruments either fail to automate the identification process fully or have low performance and need suitable leukocyte data sets for further study. To improve the current status, we need to develop more intelligent strategies. This paper investigates fulfilling high-performance automatic detection for leukocytes using a deep learning-based method. We established a new dataset more suitable for leukocyte detection, containing 6273 images (8595 leukocytes) and considering nine common clinical interference factors. Based on the dataset, the performance evaluation of six mainstream detection models is carried out, and a more robust ensemble model is proposed. The mean of average precision (mAP) @IoU = 0.50:0.95 and mean of average recall (mAR)@IoU = 0.50:0.95 of the ensemble model on the test set are 0.853 and 0.922, respectively. The detection performance of poor-quality images is robust. For the first time, it is found that the ensemble model yields an accuracy of 98.84% for detecting incomplete leukocytes. In addition, we also compared the test results of different models and found multiple identical false detections of the models, then provided correct suggestions for the clinic.

## Introduction

It is of great significance for clinicians to recognize peripheral blood leukocytes through blood smears for diagnosing leukemia, and the automation of this process can be a great help in the clinic. Peripheral leukocytes consist of five types: lymphocytes, eosinophils, neutrophils, monocytes, and basophils. Wright’s staining of blood smears is one of the standard methods for detecting leukocyte aberrations^1^, which can suggest a possible leukemia diagnosis. A rapid finding of any deviations in leukocyte populations is significant for the clinical detection of Burkitt lymphoma and acute promyelocytic leukemia because it facilitates rapid diagnosis and timely treatment^1^. However, this is a complicated, time-consuming, laborious, and subjectively influenced work for a doctor. At the same time, the clinical doctor is required to have sufficient experience^2^. Therefore, it is highly demanded to develop an automatic detection of peripheral blood leukocytes with high accuracy.

In the past, the research community and medical industry have attempted to automate the detection of leukocytes, and this automation has become a developmental trend in medical examination for blood cells^3^. Several automated cell morphology systems are based on traditional machine learning methods in the medical industry. For example, Cella-Vision^4^ fulfills some automation with digital imaging technologies, and MED-ICA EasyCell® Assistant^5^ uses image processing and pattern recognition technologies. Compared with clinical experts, although these methods provide helpful assistance and can accelerate the process of recognizing blood cells, their performance is still far behind the human experts’ level, and they cannot reliably work independently^6,7^.

Advanced automatic detection methods are primarily based on data-driven artificial intelligence (AI) using smear photographs as prior knowledge. Recent deep learning technology showed a promising solution in medical image application^8^. Since then, many publications^9–13^ have reported that the Convolutional Neural Networks (CNN) model, i.e., deep learning, is competent for image recognition tasks in different areas. Thanks to the unified, homogenous model of CNN, making use of it avoids the disadvantages of multi-step traditional machine learning methods. Given large amount of data, deep learning methods are in theory better performing than the traditional machine learning, and research community has shifted their focus to improving the use of the latest CNN architectures^14,15^. Recently, other studies have improved algorithms from different aspects and improved the accuracy of identifying leukocytes. However, these works^16–18^ focus on leukocyte classification rather than detection. Some restrictive assumptions are imposed on the classification task and the dataset. Leukocyte classification requires that the segmentation must have been done, the existence of target cells in the image must be guaranteed, and the photos mostly contain only one leukocyte. This segmentation breaks the automatic process and causes inconvenience in practical clinical applications.

Some recent research efforts have focused on leukocyte recognition as multi-object detection. Compared with leukocyte classification, the multi-object detection method drops the classification task's previous restrictive assumptions. It can automatically locate the objects and determine their types. The degree of automation is higher in multi-object detection: the quantity, locations, and types of leukocytes can be obtained simultaneously. However, the current research on leukocyte detection still needs to be improved. There needs to be more than the most existing public leukocyte datasets to support the development of leukocyte detection^19–28^. The images in existing datasets contain only one leukocyte, which is more suitable for leukocyte classification. Moreover, the current research on the detection of leukocytes uses a relatively single type of leukocyte in the dataset, and it is difficult to evaluate the level of recognition of the five types of leukocytes^29^. Most of the existing datasets are collected from one data source, and the heterogeneity of data centers is not considered^30,31^. The heterogeneity of multi-center data makes the images contain various interference factors in different distributions, which may directly affect the performance of the detection algorithm. Ragab et al.^32^ used the wiener filtering technique to purify the original data image to improve the image contrast and then conducted model training and testing. Bahaddad et al.^33^ used the ISFO algorithm to select the optimal feature subset to enhance the accuracy of the classifier. We tried to fundamentally solve the problem of multi-center heterogeneity by establishing the datasets containing 9 interference factors to strengthen the trained model’s robustness.

In this work, we collected data from multiple hospitals and established a dataset suitable for detecting leukocytes for the first time, which considered the nine interference factors that are likely to affect the performance of the detectors in detecting leukocytes^34^ in an attempt to fundamentally solve the multi-center heterogeneity problem. Based on the dataset, we tested the performance of six mainstream detection models and then tried to propose a new and more robust model using an ensemble scheme. The main contributions of this paper are summarized as follows:

1. A dedicated, diverse dataset for leukocyte detection is built up. During the collection of data samples, we considered the interference factors in practice so that the deep learning models built on these data are more robust to common interferences. The dataset is made public for researchers for further investigation.
2. Based on the dataset, the performance evaluation of six mainstream detection models is carried out, and a more robust ensemble model is proposed.
3. The proposed approach further considers and tries to address the multi-center heterogeneity problem, which is a critical problem in applying automatic leukocyte detection.

## Materials and methods

### Working pipeline of building up an AI-based detector

To apply the AI-based detection method to the automatic identification of leukocytes, the working pipeline of constructing a deep learning-based detector consists of 4 stages: (1) data preprocessing; (2) model training; (3) inference; and (4) evaluation.

#### Data preprocessing

The proposed leukocyte dataset is preprocessed in two different data formats using the dataset conversion toolbox developed by the authors. The processed dataset includes ground truth labels and split subsets in VOC and COCO format. The dataset in two formats allows us to be easily input into and trained with popular machine learning methods. The dataset also provides two sizes for all leukocyte images: (1) original size of 3264 × 3264, and (2) reduced size of 600 × 600 (mini size). The data in the former larger size can be used for error analysis, visual inspection or confirmation, and even further investigation. The data in the latter size can be used for model training, which helps to save a lot of CPU overhead time.

#### Deep learning models

The deep learning model^35,36^, i.e., convolutional neural networks, is a kind of statistical machine learning models that use convolutional operations to process input data and predict targets in a feedforward fashion. It is proven that deep learning models are capable in fitting information between different data domains. In the leukocyte detectors, we can use deep learning models to fit the medical images onto the leukocyte types and locations in the images.

A typical deep learning-based detector generally consists of two main parts: a feature extraction backbone and a regression head. The feature extraction backbones compute deep features from the image by feedforwarding the information flow through multiple convolutional layers. These deep features are vectors in the same length. The subsequent regression heads project the deep features from previous stage onto the detection output, i.e., confidence values, locations, and types. A key design requirement for the backbone networks is the isometric mapping. Namely, the backbone networks should be able to extract deep features that maintain similar distances in feature space with certain distance metric as the visual distances by human perception. The R-CNN based models^37–40^ share similar backbones, while FSAF^41^ and FCOS^42^ used Retinanet as their backbone. The regression heads for different models generally have diverse structures or complex designs, but the aim of design is the same: to better use deep features and learn the distributions of the bounding boxes and the types of target objects.

By the different structures of regression heads (with or without region proposal components), the model can be further classified into one-stage detectors^41,42^ or two-stage detectors^37–40^. In general, the one-stage detectors are running faster than the two-stage detectors, while two-stage detectors, which are more complex in general, generate more accurate predictions.

#### Model training

Using the training set, we build a deep learning-based detector with leukocyte recognition capability. Training samples with ground truth labels are iteratively input into the training algorithm in mini-batches. The models output the predicted results, compared with the ground truth labels. The training process can be stopped when the training loss curve becomes smooth, and the loss value no longer decreases in training. Finally, the internal parameters of the trained models are fixed and can be used in detecting leukocytes in new image samples from clinical practice. We set the training hyper-parameters the same to make the training process consistent for the six models.

#### Inference

In the inference stage, the detectors judge new image samples they have never seen before. New image samples collected from the clinical practice are rescaled to the input size for the trained detectors. The inference output from the leukocyte detectors includes three data items, the predicted types of leukocytes, the confidence values, and the locations of the leukocytes in the image. In our proposed method, the ensemble scheme is based on the trained detectors, optimizes the results from the results of other detectors, then outputs the final results.

#### Evaluation

In the evaluation stage, the inferred results are evaluated with multiple metrics, and the model’s performance is analyzed with different criteria. Evaluating metrics include mAP and mAR under the different values of IoU. Besides, we record executive performance, i.e., the model’s size and the inference speed in frame per second (FPS), which helps evaluate whether the detectors are suitable or feasible to put into practice. As for accuracy, we emphasize the mAP because it is a popular and proven performance indicator in object detection. To further analyze the classification capability, we measure the average precision for each type of leukocyte.

### Ensemble scheme for deep learning-based models

In machine learning, an ensemble model is a voting scheme that combines the predictions from multiple other models. The ensemble model’s work is similar to the collective judgment by a medical expert panel in diagnosing a complicated case. The advantage of an ensemble model is that the final results are more stable for complex samples and potentially more accurate in quantitative evaluation. On the other hand, however, it may cost more computational time in inference. In this work, we integrate the ensemble scheme into the inference stage and evaluate its results. Ensemble linearly combines the bounding boxes of leukocytes with the corresponding confidences as the weights^43^. Given a list of $$N$$ overlapping predicted bounding boxes $$\left\{ {\overset{\lower0.5em\hbox{$\smash{\scriptscriptstyle\rightharpoonup}$}} {L} ^{i} = \left[ {x_{1}^{i} ,{\text{~}}y_{1}^{i} ,x_{2}^{i} ,y_{2}^{i} } \right]} \right\}$$ for leukocytes and the corresponding confidence values $$\{{C}^{i}\}$$ from $$T$$ models. $$T$$ is the number of models which make most predictions on the leukocyte to be the same type. The number $$T$$ is less than or equal to $$N$$, because of possible false negative detection; and greater than $$N/2$$ because these might be some false positive detection. The averaged bounding box $$\overset{\lower0.5em\hbox{$\smash{\scriptscriptstyle\rightharpoonup}$}} {L} ^{\prime } = \left[ {x_{1}^{\prime } ,{\text{~}}y_{1}^{\prime } ,x_{2}^{\prime } ,y_{2}^{\prime } } \right]$$ and the updated confidence values $${C}^{\mathrm{^{\prime}}}$$ are given as:

$$\left[ {x^{\prime}_{1} ,y^{\prime}_{1} ,x^{\prime}_{2} ,y^{\prime}_{2} } \right] = \frac{{\sum\nolimits_{1}^{N} {\left( {C^{i} \cdot \left[ {x_{1}^{i} ,y_{1}^{i} ,x_{2}^{i} ,y_{2}^{i} } \right]} \right)} }}{{\sum\nolimits_{1}^{N} {C^{i} } }},$$

and

$$C^{\prime} = \frac{{\sum\nolimits_{1}^{N} {C^{i} } }}{T}.$$

The former equation computes the averaged location by considering the confidences of predictions from different models. A model needs to be more confident about its prediction so that its vote is more potent in influencing the final result. The latter equation suggests that the updated confidence values are averaged over the number of models rather than the number of predictions. In this way, if some models deem that the area has no leukocyte, like abstention, the updated confidence values will be lowered.

**Figure 1:**
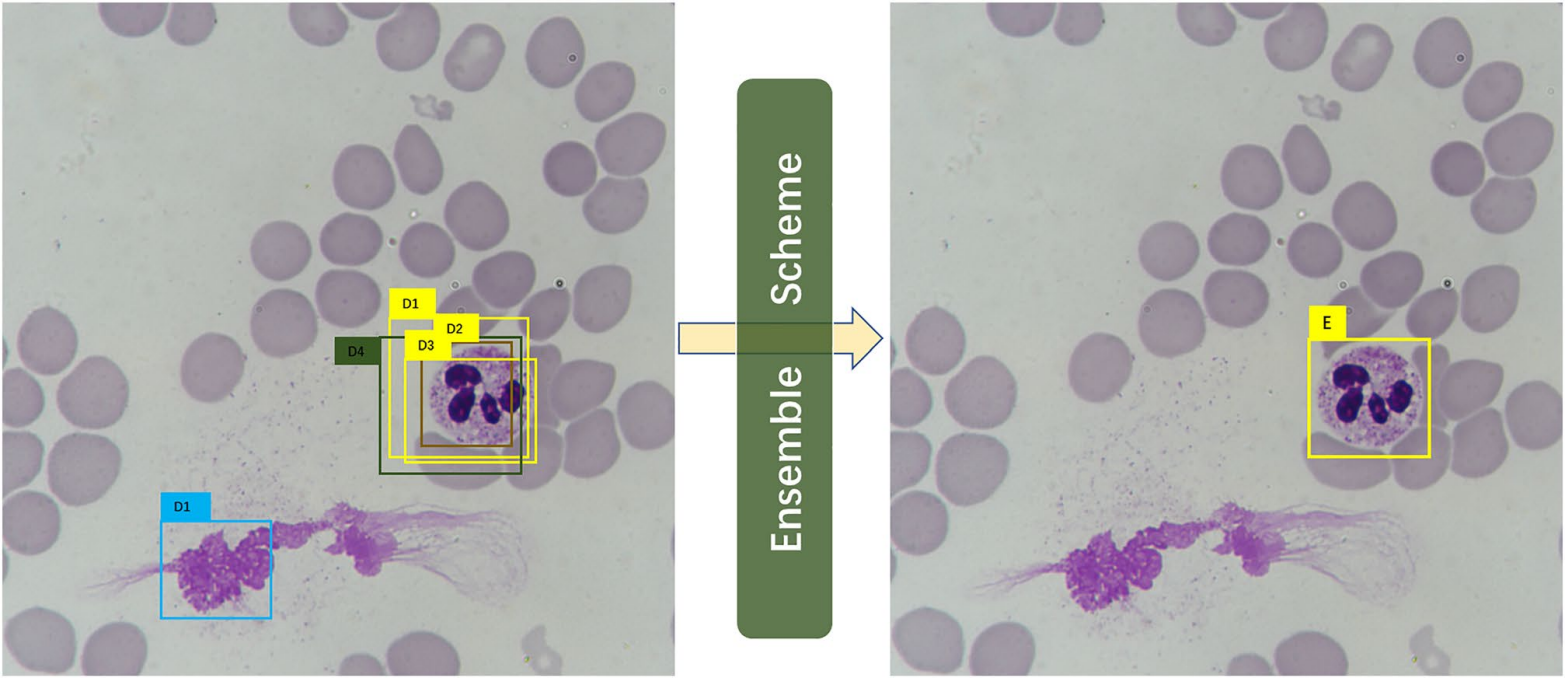
An illustrative example of the ensemble scheme. The raw detections are given in the left image, and the right image is the result after ensemble.

A simple illustrative example is shown in Fig. 1. Suppose we have trained six models and five out of six output the 5 detections as *D1*, *D2*, *D3*, *D4*, and *D5* in the left image in Fig. 1. Each *Di* contains a bounding box location $$\overset{\lower0.5em\hbox{$\smash{\scriptscriptstyle\rightharpoonup}$}} {L} ^{i}$$, a confidence value $${C}^{i}$$, and a type label *P*^*i*^. In the ensemble process, if the number of overlapping detections for a possible object is less $$N/2$$, these detections will be discarded in the first place. Thus, in this example, D1 is ruled out, leaving *D1*, *D2*, *D3*, and *D4* for further processing. Three (D1, D2, D3) out of the four detections predict leukocyte as neutrophil granulocyte, and then the ensemble scheme judges the type of ensemble prediction *E* as the same as that of the majority. The ensemble scheme subsequently calculates the confidence value and the bounding by computing the corresponding mean values.

### Implementation details

The experiments are implemented on a regular workstation computer with Intel Core i5-8600 CPU, 16 GB RAM, and an Nvidia TITAN Xp Graphics Card with 12 GB graphic memory. The software environments are based on Ubuntu 18.04 OS, and training is carried out on PyTorch 1.7.0 (https://pytorch.org/) and mmdetection 2.6.0 codebases (https://github.com/open-mmlab/mmdetection). The training epoch is set to 16, which is high enough for training convergence for the detectors. Setting this relatively excessive number of epochs is to ensure the models can approach the optimal state and avoid underfitting. The recorded training time for the models is around two hours.

The models can be efficiently trained in such a short time because of the use of pre-trained backbone networks and fine-tuning techniques. The fine-tuning technique allows the model to shift its intelligence from recognizing generic objects to detecting leukocytes. The pre-trained weights are trained parameters from a deep learning-based model (Resnet-50^44^) in classifying objects in the ImageNet dataset or detecting objects in the MS-COCO dataset. To fine-tune a model, we freeze the parameters in the low-level filters, which compute basic image texture features. On the other, high-level parameters, which are, for a reason, the structural information, are gradually updated by the backpropagation approach. This way, it adjusts the high-level parameters in pre-trained weights to our leukocyte detection task.

When training detectors, we used stochastic gradient descent (SGD) as the optimizer for the model parameter update. The key hyper-parameters, i.e., the learning rate, momentum, and weight decay coefficient of SGD, are set to 0.01, 0.9, and 0.0001, respectively. Other detailed configurations for the detector architecture are defaults except for these settings.

Data augmentation technique is employed in model training. Data augmentation is an online process that dynamically generates variants of training samples before feeding them into the model, following the sampling of the mini-batch of training data from the training set. Supplementary Table 1 shows a transformation list of data augmentation used in our implementation. The transforms are composed of an occurrence probability for each transform.

### Ethical approval

All blood smears involved in this study are historical samples. Since only blood smears from patients are photographed, the approval of the institutional review board is not required.

## Results

### Establishment of a dataset with interference factors

The 111 Wright-stained blood smears were collected from Tianjin Medical University Affiliated Medical Center (Tianjin Cancer Hospital and Tianjin Children's Hospital) and the Rehabilitation Hospital of Hexi District, Tianjin. Five types of leukocytes from each smear were photographed by a Nikon DS-Ri2 Color Camera at × 1000 original magnification for analysis. The five types of leukocytes include neutrophil (NG), basophil (BG), eosinophil (EG), lymphocyte (L), and monocyte (M). Subsequentially, 6273 images in total were obtained, with nearly 2000 images containing multiple leukocytes, which is the first dataset suitable for leukocyte detection. These leukocyte images were divided into the training set and the test set with a ratio of 4:1.

**Figure 2:**
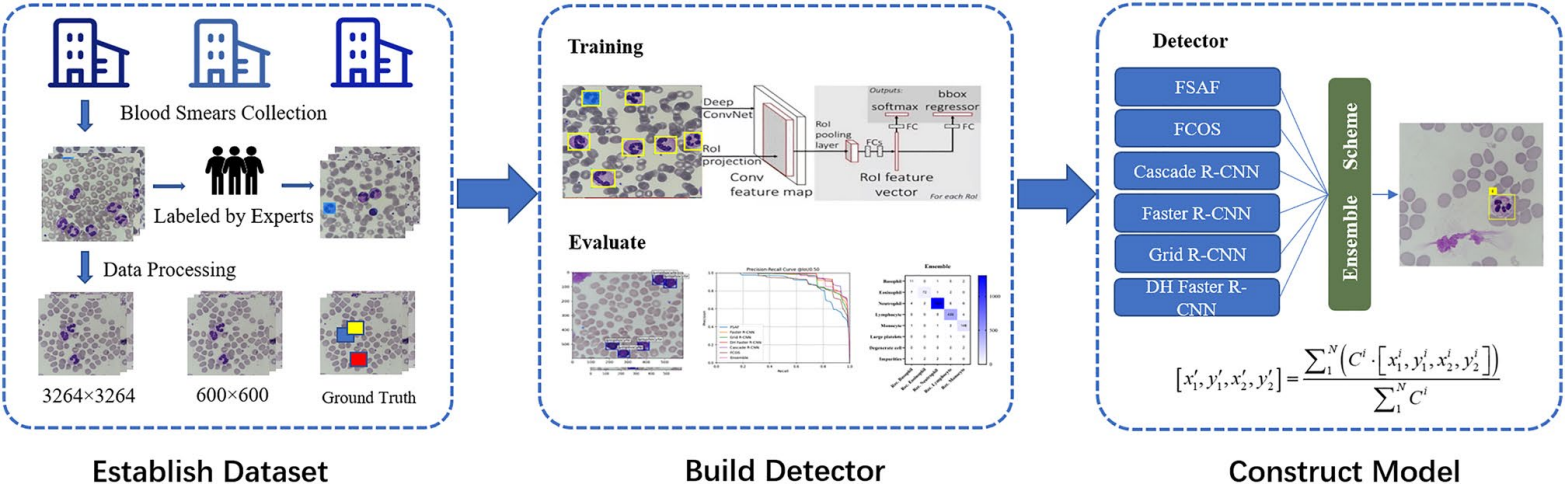
The flowchart of model training and evaluation.

Each sample in the dataset contains two items: a visual signal map in the form of a color image that may contain more than one type of leukocyte and a manually labeled ground truth indicating the location(s) and type(s) of existing leukocyte(s). The ground truths are separately annotated by three experts with 18-year clinical experience using Labellmg toolkit software. During expert review and confirmation of the data samples, any cells with inconsistent type labels from different experts will be taken out. However, when the top-3 model made the same mistake, recognizing the same cell as another cell, we invited another expert with more experience to review and verify the data labeled by the above three experts. To avoid the problem of data imbalance, we have narrowed the statistical distribution gap among the five types of leukocytes as much as possible. Table 1 shows the composition of the five types of leukocytes in the training set and the test set. The processes of dataset formulation are also depicted in the corresponding part of the flowchart presented in Fig. 2.

**Table 1:** The number of images and leukocytes in the created dataset.

Sources	Types and sample numbers					
	Neutrophil images (cells)	Basophil images (cells)	Eosinophil images (cells)	Lymphocyte images (cells)	Monocyte images (cells)	Total images (cells)
Training Set	1214 (2812)	282 (286)	1006 (1018)	1232 (1440)	965 (1031)	4699 (6587)
Test Set	1015 (1323)	12 (14)	71 (82)	355 (439)	121 (150)	1574 (2008)
Total	2229 (4135)	294 (300)	1077 (1100)	1587 (1879)	1086 (1181)	6273 (8595)

**Figure 3:**
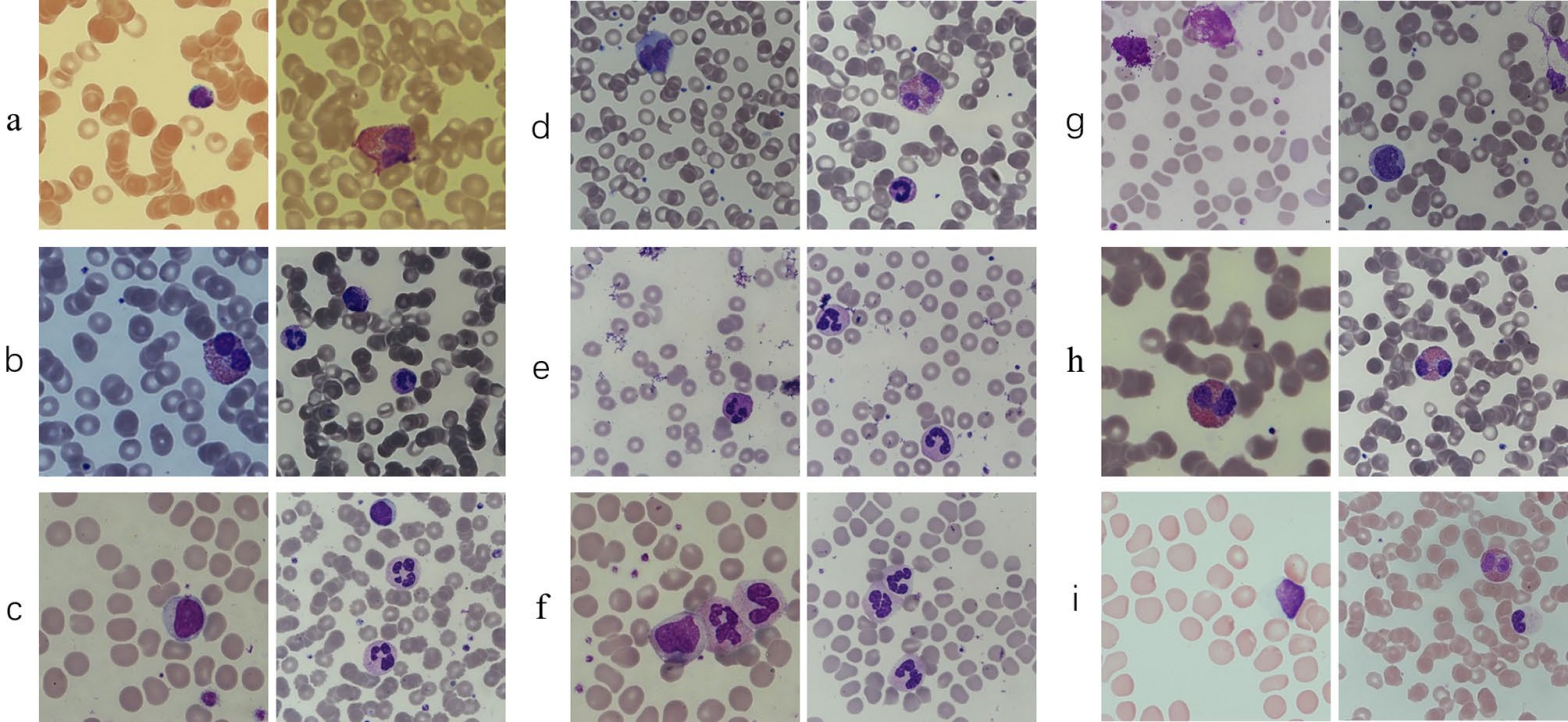
Some exemplar samples are affected by the interference factors. (**a**) Colour casts on blood cell smear images; (**b**) low illuminative intensity of blood cell smear images; (**c**) giant platelets; (**d**) incorrect imaging focal length of cell images; (**e**) images containing dyes or other impurities; (**f**) overlapping leukocytes; (**g**) degenerated leukocytes; (**h**) excessively high phosphate buffer solution (pH > 6.8); (**i**) excessively low phosphate buffer solution (pH < 6.4).

Some “worse cases”, which are likely to appear in practical clinical scenarios, should be included in the AI model’s prior knowledge (training set) for assisting in the detection of leukocytes. The dataset we created contains nine factors that interfere with leukocyte detection, making the trained model generalize well. Some typical cases are shown in Fig. 3. The statistics of the interference factors are manually collected and summarized. The specific numbers of these situations in the training set are shown in Supplementary Table 2. Meanwhile, these images contain multiple leukocytes, making the dataset more suitable for detection research. The statistical summaries of our dataset are presented in Supplementary Table 3.

### Performance comparison of the six models and ensemble model result on test sets

**Figure 4:**
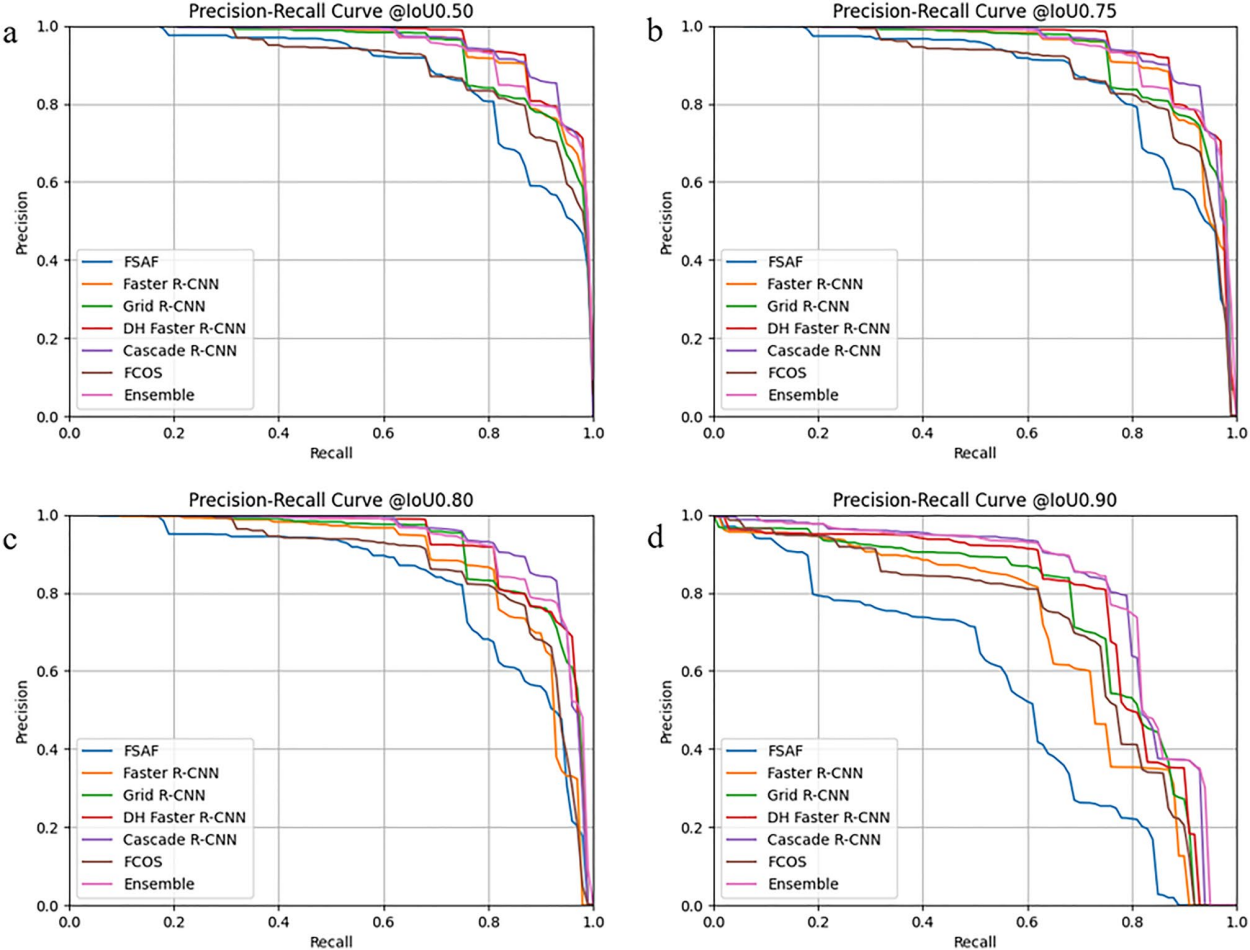
The PR curve of each model at different IoU. Performance comparison of the six models and ensemble model on test sets. The x-axis represents the recall values, and the y-axis represents the precision.

We use the test set to evaluate the six well-trained detection algorithm models and draw each model’s the precision-recall (PR) curve under different IOU thresholds, as shown in Fig. 4. In Fig. 4, it is not difficult to find that when the IOU threshold is lower than 0.8, all models output good results, and the AUCs are quite large. Among them, cascade RCNN and ensemble model are the TOP two among their competitors, FSAF is on par with other counterparts, and other models have average performance. When the IOU threshold is increased to 0.9, this means that the criteria for successful detection are more stringent. We can see that the divergence of the curves from each other increases. Now we can more easily distinguish the difference in performance. The advantages of cascade RCNN and ensemble model are more prominent. The corresponding specific quantitative results are shown in Table 2. For the test set, mAP@IoU = 0.50:0.95 of cascade RCNN is higher than the ensemble model (0.856 > 0.853), but its mAR@IoU = 0.50:0.95 is 0.909, which is lower than that of the ensemble model (0.909 < 0.922). In addition, the Top 2 models have the best recognition performance on NG, and AP@IoU = 0.50:0.95 is 0.916 and 0.925, respectively. The performance of BG recognition is low; AP@IoU = 0.50:0.95 is 0.781 and 0.752, respectively. Table 2 also shows the corresponding detection performance indicators of other detection models.

**Table 2:** Performance in key evaluation criteria of six methods and ensemble model on the test set.

Model	mAP	mAP @IoU0.50	mAP @IoU0.75	mAR	AP-BG	AP-EG	AP-NG	AP-M	AP-L
FSAF	0.742	0.874	0.860	0.866	0.618	0.684	0.870	0.756	0.785
FCOS	0.795	0.896	0.880	0.913	0.642	0.826	0.894	0.795	0.819
Faster R-CNN	0.815	0.940	0.919	0.879	0.738	0.847	0.893	0.785	0.814
Grid R-CNN	0.822	0.926	0.920	0.903	0.709	0.836	0.847	0.890	0.832
DH Faster R-CNN	0.848	0.951	0.939	0.910	0.753	0.892	0.908	0.842	0.843
Ensemble	0.853	0.940	0.933	0.922	0.752	0.898	0.925	0.843	0.848
Cascade R-CNN	0.856	0.948	0.938	0.909	0.781	0.905	0.916	0.838	0.841

In this work, we also try to improve the performance by integrating an ensemble model as a post-process for the results. Although its mAP@IoU = 0.50:0.95 is slightly lower than the Cascade RCNN among the tested models (0.853 < 0.856), its mAR@IoU = 0.50:0.95 is the highest (0.922 > 0.909), which means that the ensemble model has the lowest rate of missed detection of leukocytes (Fig. 4). That helps count leukocytes and prompts experts to verify the model to detect the wrong leukocytes. In addition, for the detection of leukocyte subtypes such as NG, M, and L, the ensemble model performs best, surpassing the cascade RCNN in the current evaluation models shown in Table 2.

### The performance comparison of detecting leukocytes with Cascade R-CNN and ensemble model

**Figure 5:**
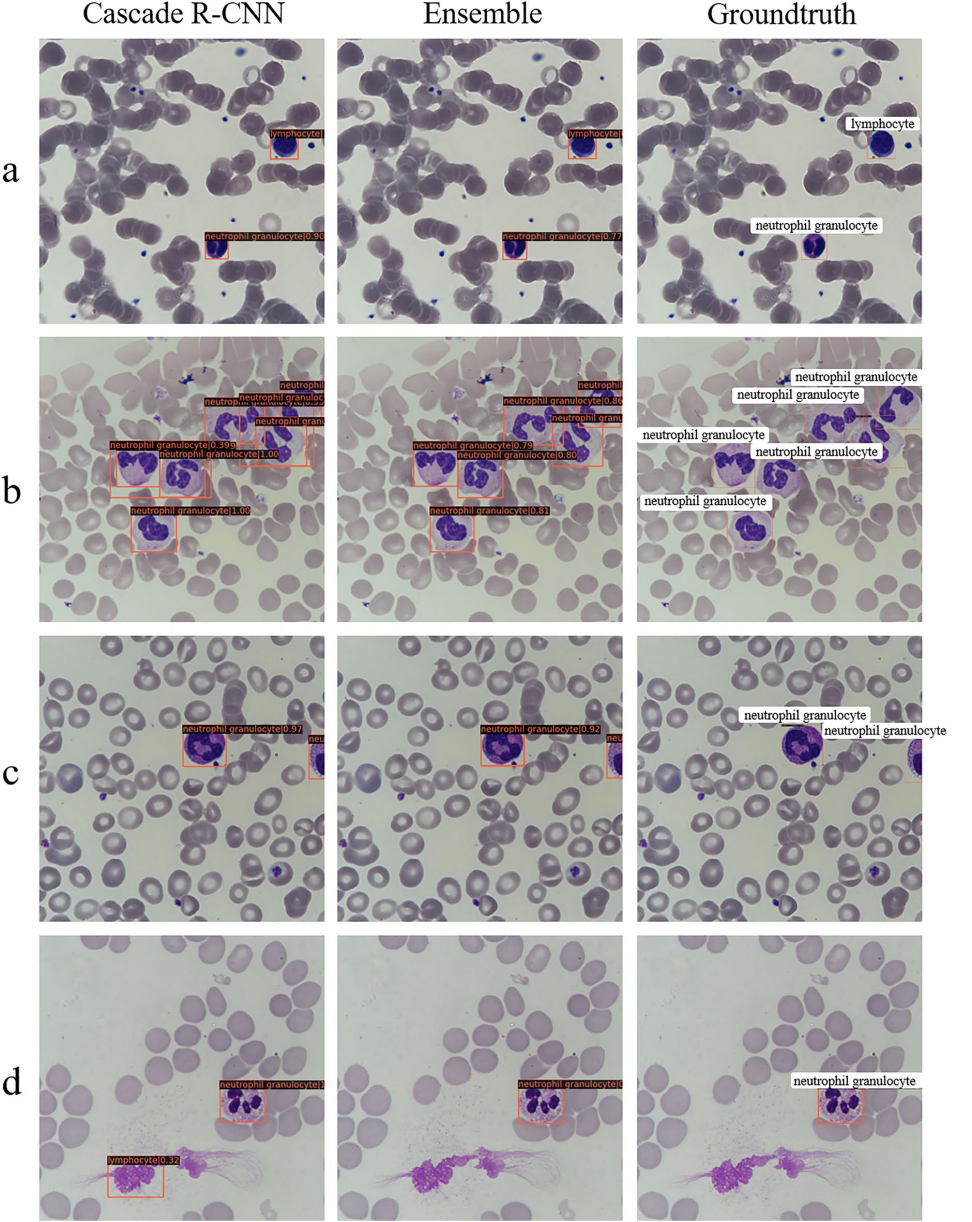
Some real examples of detecting leukocytes in different scenarios with Cascade R-CNN and the ensemble model. (**a**) An example of excessively high phosphate buffer solution (pH > 6.8) and small neutrophils. (**b**) An example of overlapping leukocytes. (**c**) An example of incomplete leukocytes. (**d**) An example of impurities in the picture.

Detection of images is always challenging due to possible variance in staining, overlapping leukocytes, impurities, or even incomplete leukocytes. We deliberately considered these factors that easily affect the performance of the detection model on the dataset. In the test set, we focused on the detection effects of the Cascade R-CNN with the highest mAP values and the ensemble model. The accuracy of detecting leukocytes is 92.17% and 95.30% on the overly stained 447 images, respectively. Figure 5a shows the detection results of some images stained heavily. In addition, the detection accuracy of Cascade R-CNN and the ensemble model are 65.79% and 94.74% for the detection of overlapping leukocytes, respectively. Figure 5b shows the detection performance of the model on leukocyte-dense scenes. From the results, the detection performance of Cascade R-CNN for dense scenes needs to be further improved. In the test set, there are 170 pictures containing impurities such as dye residues, cell debris, etc. The Cascade R-CNN and the ensemble model can better eliminate impurities when detecting leukocytes. The ratio of the impurities mistaken for leukocytes is 8.23% and 3.53%, respectively. Figure 5c shows the result of the model's detection of leukocytes containing impurities. In addition, the more surprising point is that the two models can accurately determine the type and location of incomplete leukocytes. The detection accuracy of incomplete leukocytes is 97.67% and 98.84%, respectively. Figure 5d shows the detection results of the model on incomplete leukocytes. Supplementary Table 4 shows the specific number of correct detections.

**Figure 6:**
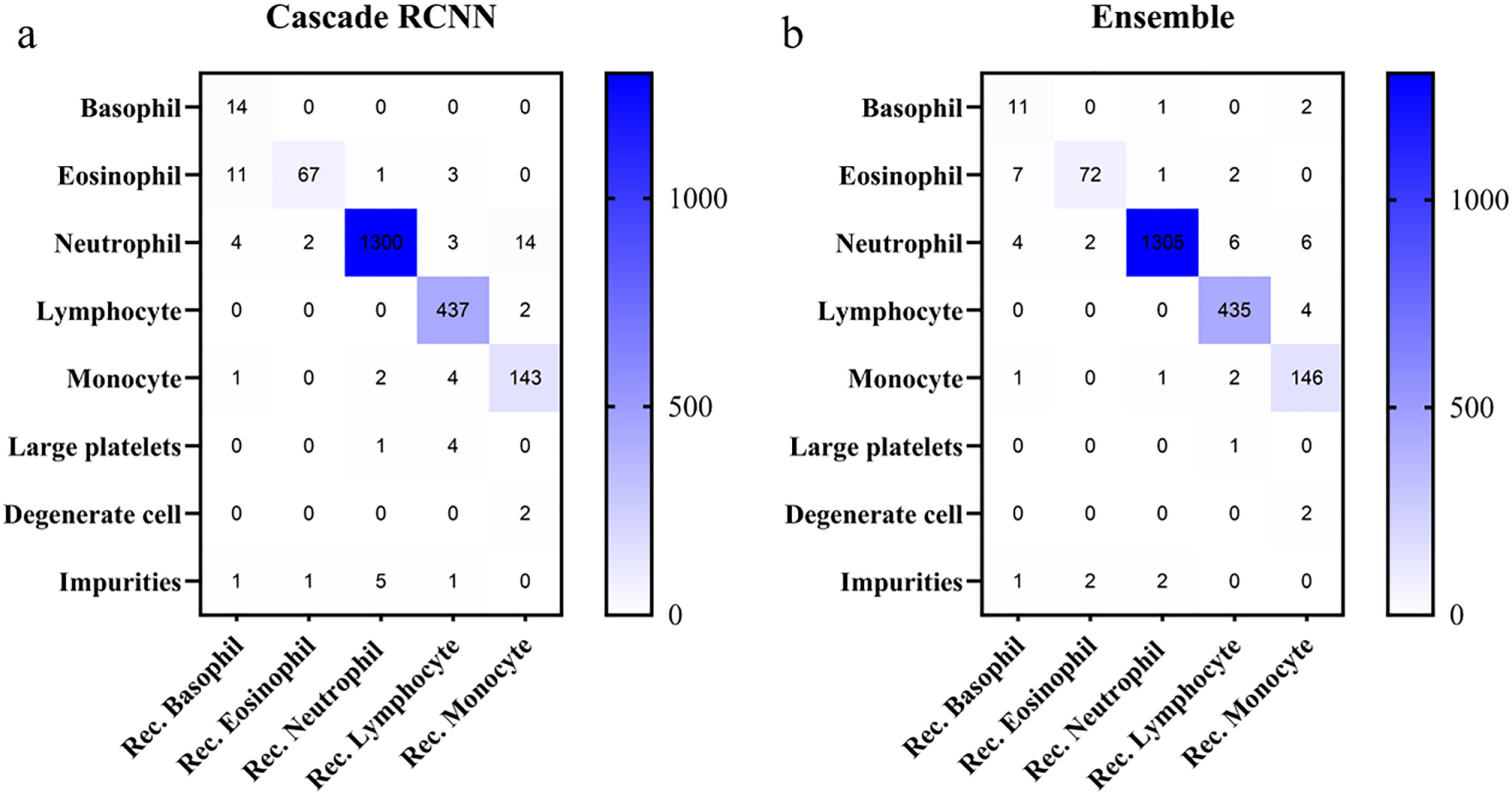
The heat map of the confusion matrix for the Cascade R-CNN and the ensemble model’s performance detecting five types of leukocytes on the test set.

To further investigate the model’s capabilities, we analyze the classification performance of Cascade R-CNN and the ensemble model comparatively. The confusion matrix of Cascade R-CNN and the ensemble model is shown in Fig. 6. In Fig. 6a, we found identifying eosinophils with Cascade R-CNN challenging, and some neutrophils are mistaken for monocytes. Meanwhile, the accuracy of eosinophils or basophils was relatively lower than other types because many eosinophils were misclassified into basophils. It is difficult for the ensemble model to identify basophils and eosinophils, and a small part of neutrophils are incorrectly identified as other types of leukocytes (Fig. 6b).

## Discussion

In this study, we established a dataset with multi-leukocyte images, considering nine common interference factors in clinical application. Based on the research foundation and search results of the machine vision algorithm for detecting blood cells, we selected six detection models: Cascade R-CNN, DH Faster R-CNN, grid R-CNN, Faster R-CNN, FCOS, and FSAS for leukocyte detection. Cascade R-CNN has the best detection performance, mAP@IoU = 0.50:0.95 is 0.856, and mAR@IoU = 0.50:0.95 is 0.909. Then we provide a powerful ensemble model. Without major modifications, the ensemble model can obtain high-performance indicators for leukocyte detection. mAP@IoU = 0.50:0.95 is 0.853 and mAR@IoU = 0.50:0.95 is 0.922. Through further in-depth analysis of the detection performance of Cascade R-CNN and the ensemble model, it is found that the ensemble model may be a better choice for the automated blood cell morphology system.

Datasets are the basis for solutions using data-driven artificial intelligence. The existing public leukocyte datasets^19–28^ are either small data, or the images only contain a single leukocyte. These datasets are considered simple and cannot support the building up of an intelligent model for the complicated scenario in the clinic. To our knowledge, this study created the first dataset with multi-leukocyte images close to the practical environment of clinical testing of peripheral blood smears. As shown in Fig. 3, the dataset considers nine frequent interference factors in the clinical blood cell recognition process. The image is not limited to one leukocyte but includes multiple leukocytes, which is more suitable for the clinical environment. Considering these interference factors in the dataset and using the online data augmentation technique, the Cascade R-CNN and other models were trained on much broader data distributions, increasing generalization and alleviating the multi-center heterogeneity problem.

Research on using AI to detect leukocytes is still limited in the literature. A report showed that mAP@IoU = 0.5 used to detect normal leukocytes was 0.931^30^. Fanous et al.^45^ first converted the SLIM images into bright-field micrographs using the AI model, then performed the parallel task of locating and labeling cells using the object detection model EfficientNet and finally achieved an average accuracy of 75% in locating and classifying neutrophils, eosinophils, lymphocytes, and monocytes. Alhazmi^46^ utilized the BCCD dataset to train deep learning models to detect leukocytes, red blood cells, and platelets, thus counting them, but did not further classify the types of leukocytes. However, when we evaluate the performance of the detection model, we consider common clinical interference factors, which increase the difficulty of detection and are closer to the actual clinical detection environment. The mAP@IoU = 0.5 of the Cascade R-CNN and ensemble model are 0.948 and 0.940 on the test set, higher than 0.931^28^ and 75%^45^ (Table 2). In addition, although the mAP@IoU = 0.50:0.95 of the ensemble model is slightly lower than that of Cascade R-CNN (0.853 < 0.856), its mAR@IoU = 0.50:0.95 is the highest (0.922 > 0.909), which means the integrated model has the lowest rate of missed detection of leukocytes (Fig. 4). It helps to calculate leukocytes and prompts experts to verify the model to detect wrong leukocytes. In addition, for detecting leukocyte subtypes such as NG, M, and L, the ensemble model performs best, surpassing the Cascade R-CNN model, as shown in Table 2.

The performance of the two models is further analyzed from the results of the challenging cases. Both Cascade R-CNN and the ensemble model are robust to significant pH changes beyond the normal pH range [6.4, 6.8], and the models can accurately locate and identify poorly stained leukocytes. The ensemble model performs better (95.30% > 92.17%) (Fig. 5a). Moreover, the detection ability of the ensemble model in the dense scenes is also higher than that of Cascade R-CNN, with a detection accuracy rate of 94.74% > 65.79%, which makes the potential model advantages in the detection of some leukemias (Fig. 5b). In the clinical detection of leukocytes, common impurities, including dye residue, broken red blood cells, dust, etc., frequently occur. These common impurities will affect the performance of the detection model. Cascade R-CNN and the ensemble model are robust to the interference of impurities. The latter performs better and has a low probability of misjudgment of impurities as leukocytes (3.53% < 8.23%) (Fig. 5c). It is worth noting that for the first time, this article found that both Cascade R-CNN and the ensemble model can detect the types of incomplete leukocytes with high accuracy^29,30,47^. The ensemble model is slightly better than Cascade R-CNN (98.84% > 97.67%), even for only covering 25–50% of the cells in a limited visible area, which means that the algorithm is superior to traditional image recognition algorithms (Fig. 5d).

**Figure 7:**
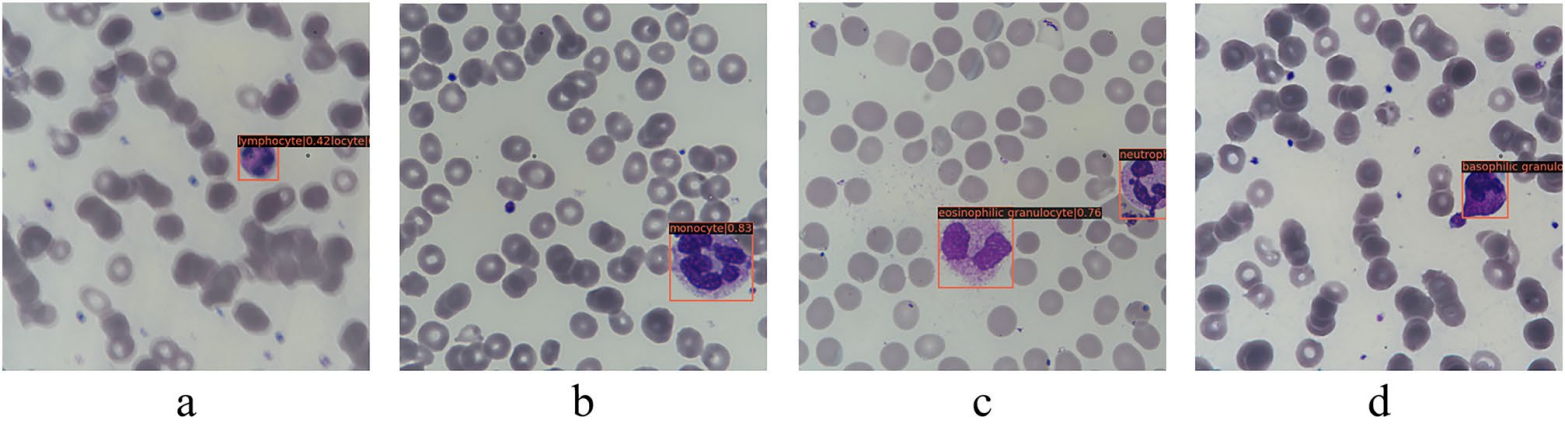
Some examples of partial neutrophils misidentified by the ensemble model. (**a**)–(**d**) The neutrophils were misidentified separately as lymphocyte, monocyte, eosinophil, and basophil.

Regarding Cascade R-CNN and the ensemble model for the lowest detection results of basophils (Table 2, Fig. 6), research shows that in computer vision tasks, more training examples can improve performance indicators, and the success of image classification tasks largely depends on the availability of labeled data^29^. A total of 8595 leukocytes were collected in our study. However, the distribution of labels varies due to the different proportions of leukocyte types in the blood. There are 300 basophils in the training set and test set, while the numbers of other types of leukocytes are all > 1000. Therefore, basophils showed the lowest mAP values in several models, ranging from 0.618 to 0.781. It is expected that its detection performance will be significantly improved with the expansion of the training set in the future and the increase of basophils. Since the established dataset contains various interference factors, although the ensemble model has strong anti-interference ability, a small number of leukocytes were misidentified. For example, in Fig. 7a, the neutrophil was small and deeply stained, which was mistaken as the lymphocyte. In Fig. 7b, the neutrophil was large and had many nuclear lobes, which was identified as a monocyte. In Fig. 7c, the degenerate neutrophil was redder and had an unclear cellular structure, which was mistaken for eosinophil. In Fig. 7d, the neutrophil granulocyte was deeply stained and contained toxic particles, which was identified as a basophil. If the number of images with various interference factors can be increased in the future, the recognition accuracy of the model will be higher.

**Figure 8:**
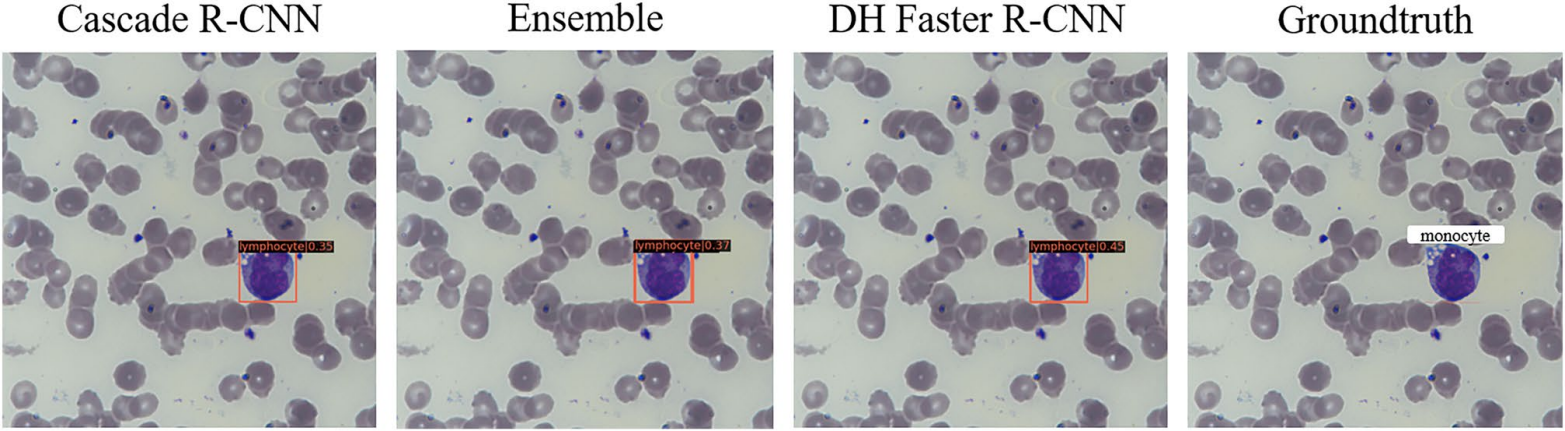
Qualitative true positive results yielded by different models. For the same leukocyte, Cascade R-CNN, Ensemble model, and DH Faster R-CNN all detect it as a lymphocyte, and experts labeled it a monocyte.

The error detection result map of the models may provide some correct suggestions to the clinical detection staff. As shown in Fig. 8, we noticed consistent detection errors among the top-three models, which all detected a monocyte as a lymphocyte. In the post-review, it was noted that the leukocyte was considered an inaccurate marker by clinical experts. The leukocyte is an atypical lymphocyte. As we know, there are three subtypes of atypical lymphocytes: plasmacyte prototype (I), monocyte prototype (II), and prolymphocyte prototype (III), where the shape of sub-type II is close to monocyte. It is difficult to accurately identify them in clinical practice, which needs to be combined with the overall blood smear of the subject and other test results to make a comprehensive judgment. Clinicians usually observe blood smears under a microscope. Since the cells are stereoscopic, they generally need to constantly adjust the fine quasi-focal spiral to observe the characteristics of the images of different layers of leukocytes, which can improve the recognition accuracy^48^. Supposing that only images of leukocytes are given to doctors, even trained pathologists cannot guarantee accurate identification of all of them, especially when they are presented with cells of similar morphology, such as many large atypical lymphocytes that look similar to small monocytes^45^. Furthermore, that reflects the correct suggestions from the model to some extent. The deep network has learned many image characteristics of leukocytes of different shapes. The model summarizes the unique features of distinguishing different types of them, which may improve the level of clinical detection of leukocytes. However, the model's ability for atypical lymphocyte detection needs to be strengthened in future work.

In the future, with the current well-established baseline, we plan to extend our work and overcome some limitations. Firstly, the current size of the dataset only meets the primary demand for data-driven deep learning. We plan to collect more data to help the deep learning models generalize better. Secondly, some data imbalance still exists in the current dataset, partly because basophil cells appear less than others. More samples of basophil will be targeted during the expansion of the dataset. The current detector is built using the large backbone models pre-trained on the 1000-class ImageNet dataset. Technically, it is possible to build up an effective detector for six types of objects by using lightweight backbone models, and in the next stage, exploring lightweight models should be the new focus. The outcome can lead to lower computational costs and more flexible deployment in embedded devices.

In summary, the ensemble model may be a better choice for automatic blood cell morphology systems. Using the ensemble model, the leukocyte detection process can reduce the dependence on experts, overcome the inherent limitations of clinicians' manual identification, such as the influence of subjectivity on identification, and improve the consistency of diagnosis. By considering the interference factors and training on collective knowledge from experts, the ensemble model “remembered” the accurate and wide-ranged prior information from the clinic. Therefore, the model is highly adaptive and robust to daily clinical environments. Another advantage of the ensemble model is that its processing speed demonstrates a tremendous advantage over manual recognition and reduces workload.

## Conclusions

Our paper established a dataset with multi-leukocyte images, considering nine common interference factors in clinical application. We evaluated six mainstream detection models and developed a new model to evaluate their performance in terms of mAP@IoU = 0.50:0.95, mAR@IoU = 0.50:0.95 comprehensively, AP for each type of leukocyte, and robustness to different interference factors. The developed ensemble model can count leukocytes more accurately and prompt experts to detect wrong detections, and it is more robust. In addition, we found that the model's error detection result can provide clinical with some correct suggestions, which can help experts perform clinical testing.

We also build a web service to test data from a different source. The web service helps increase technical readiness of our work, which reflects its potentials in deployment as well. Once the automatic leukocyte detector is fully deployed, the service can be provided in the clinic in the countryside or in remote regions, where there is a lack of well-maintained instruments and skilled medics, and help medical personnel diagnose leukocyte-related diseases.

## Supplementary Information

Supplementary Information.

## Data Availability

The datasets used and analyzed during the current study are available from the corresponding author upon reasonable request.
