# Supplementary material for: A deep learning model for detection of leukocytes under various interference factors
Source: Sci Rep. 2023 Feb 7;13:2160. doi: 10.1038/s41598-023-29331-3 (PMC9905612; doi:10.1038/s41598-023-29331-3)
Supplement: Supplementary file 1 — Supplementary Information. [file 41598_2023_29331_MOESM1_ESM.docx]

**Supplementary materials**

**Evaluation Criteria**

The main metric for evaluating the overall performance of our leukocyte detector is mean average precision (mAP) [http://mscoco.org/], which is a popular performance indictor in object detection domain. Given a set of pre-set IoU thresholds, the mAP is the mean value of average precision across all leukocyte types to be detected, which can be expressed as follows:

$\mathrm{mAP}=\frac{1}{|C|}\sum_{c\in C} {AP}_{c}$ , (1)

where $C$ is a label set of all leukocyte types in dataset, $|C|$ is the order or number of items of set $C$, and ${AP}_{c}$ is average precision across different an IoU thresholds set $U$ for leukocyte type $c$, which is a function of ${TP}_{c}^{u}$ and ${FP}_{c}^{u}$:

${AP}_{c}=\frac{1}{|U|}\sum_{u\in U} \frac{{TP}_{c}^{u}}{{TP}_{c}^{u}+{FP}_{c}^{u}}$ . (2)

Generally, the higher mAP value suggests the higher of recognition accuracy of the model for leukocytes in the image. However, the variance between APs of leukocyte types should not be too high. Otherwise, the accuracies of different types are imbalanced.

The mean average recall (mAR), which is another commonly used metric, is also employed as auxiliary indicator in our experiments. The average recall (AR) is mathematically expressed as:

${AR}_{c}=\frac{1}{|U|}\sum_{u\in U} \frac{{TP}_{c}^{u}}{{TP}_{c}^{u}+{FN}_{c}^{u}}$ . (3)

The mAR is the average recall (AR) of multiple types of leukocytes in the image, and the higher value represents the lower missed detection rate of leukocytes recognition by the model.

The specific meanings of ${TP}_{c}^{u}$, ${FP}_{c}^{u}$ and ${FN}_{c}^{u}$ are given as follows:

${TP}_{c}^{u}$: The number of true positive detections which the detected bounding boxes exhibit a higher IoU overlapping than threshold $u$. True positive detections are objects that are correctly classified as the groundtruth type $c$.

${FP}_{c}^{u}$: The number of false positive detections that come from two type of false positive cases. One type of the false positive is the detected bounding boxes that exhibit higher IoU overlappings than threshold $u$, however, are incorrectly classified into other types rather than the groundtruth type $c$. Another type of the false positive is the detected bounding boxes that contain no underlying object or merely contain parts of the objects with a lower IoU overlapping than threshold$u$.

${FN}_{c}^{u}$：The number of false negative detections, which is the number of groundtruth objects with no detection or with no covering bounding box shows a higher IoU overlapping than threshold $u$. In other words, the detector misses the targets.

A detection, regardless being correct or incorrect, is determined by whether its confidence is higher than the pre-set confidence threshold. Through all our experiments, the confidence threshold is pre-set empirically to 0.05; In the MS-COCO criteria, the IoU thresholds are a set of percentage values from the range of [0.5,0.95] with a step of 0.05; while in the more relaxed Pascal VOC criteria, the IoU is by default set to be 0.5.

**Supplementary Figures and Tables**

**Supplementary Figures**

**
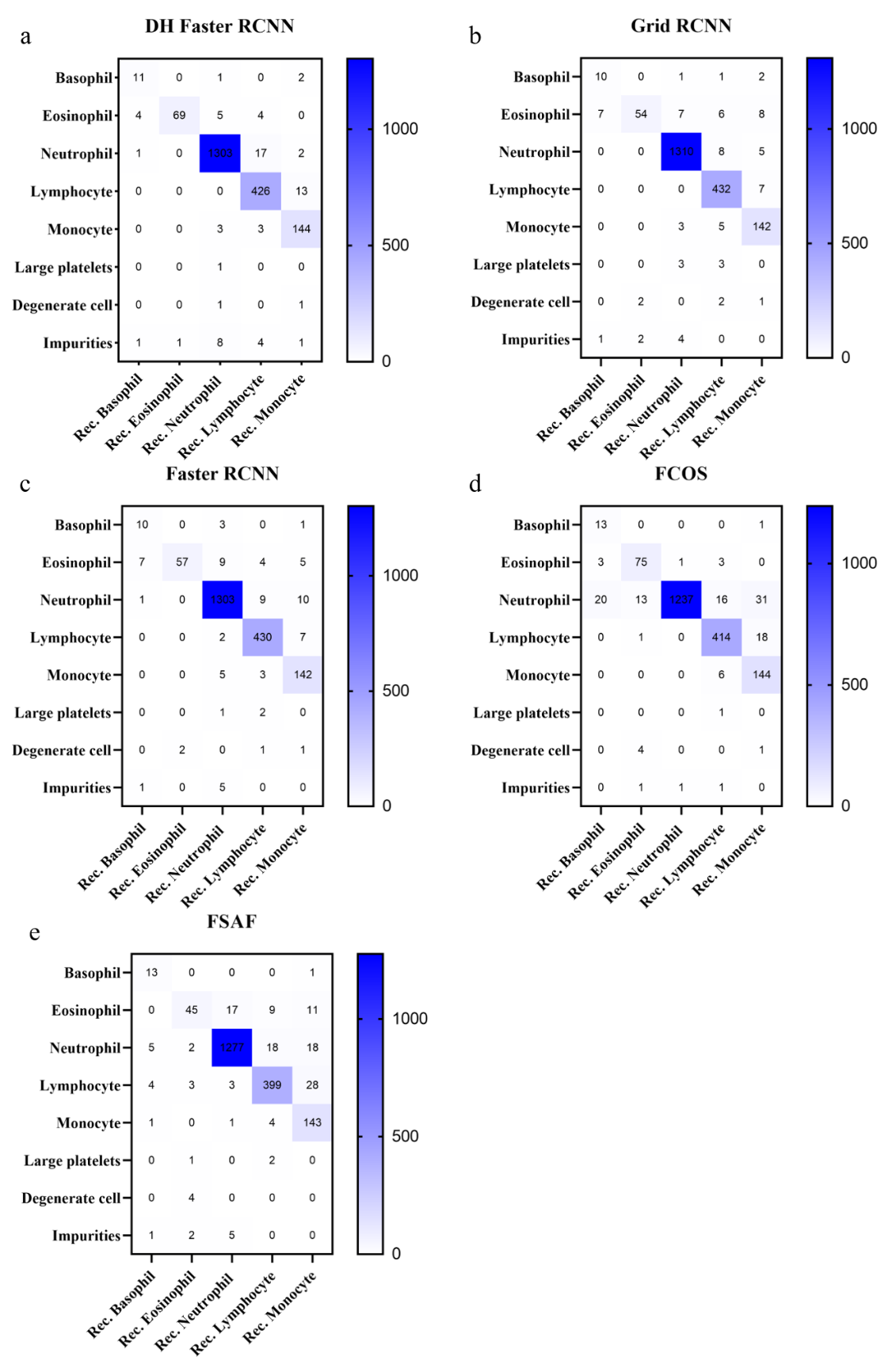
**

**Supplementary Figure 1:** The heat map of the confusion matrix illustrates the performance of the DH Faster R-CNN, Grid R-CNN, Faster R-CNN, FCOS, and FSAF in detecting five types of leukocytes on the test set.

**Supplementary Tables**

**Supplementary Table 1:** Detailed transforms list of data augmentation and their parameters.

| Type of Transform | Parameters | Occurrence Probability |
| --- | --- | --- |
| Flip | - | 0.5 |
| RandomRotate90 | - | 0.5 |
| RGBShift | 10 (Shift limit for each channel) | ~0.1 |
| HueSaturationValue | 20 (Shift limit for each channel) | ~0.1 |
| GaussNoise | - | ~0.05 |
| RandomBrightnessContrst | [0.1, 0.3] variation range | ~0.1 |
| RandomGamma | - | ~0.1 |

**Supplementary Table 2:** The number of images that interfere with leukocyte detection in our training set.

| Types | BG | EG | L | M | NG |
| --- | --- | --- | --- | --- | --- |
| Color casts on blood cell smear images | 5 | 129 | 58 | 36 | 2 |
| Low illuminative intensity of blood cell smear images | 23 | 68 | 31 | 44 | 2 |
| Giant platelets | 58 | 88 | 65 | 63 | 109 |
| Incorrect imaging focal length of cell images | 21 | 33 | 31 | 18 | 11 |
| Images containing dyes or other impurities | 47 | 109 | 269 | 217 | 246 |
| Overlapping leukocytes | 2 | 22 | 12 | 19 | 82 |
| Degenerated leukocytes | 3 | 11 | 4 | 2 | 2 |
| Excessively high phosphate buffer solution pH >6.8 | 90 | 235 | 82 | 62 | 77 |
| Excessively low phosphate buffer solution pH<6.4 | 4 | 117 | 76 | 32 | 9 |

NG: neutrophil, BG: basophil, EG：eosinophil, L：lymphocyte, M：monocyte

**Supplementary Table 3：**Multiple leukocytes of our training set.

| Types | One | Two | Three | Four | Five | Six | Seven | Ratio of multiple leukocytes |
| --- | --- | --- | --- | --- | --- | --- | --- | --- |
| Numbers | 3365 | 933 | 294 | 76 | 24 | 5 | 2 | 39.64% |

**Supplementary Table 4：** Cascade R-CNN and ensemble models correctly detect the number of pictures of leukocytes in different scenarios.

| **Models** | **pH >6.8** | **Overlapping** | **Incomplete** | **Impurities** |
| --- | --- | --- | --- | --- |
| Cascade R-CNN | 412/447 | 25/38 | 84/86 | 156/170 |
| Ensemble | 426/447 | 36/38 | 85/86 | 164/170 |

a/b: a represents the number of pictures correctly detected by the model in each scene; b represents the total number of pictures in each scene.

**Supplementary Table 5:** Hardware and Software Environments for Implementation.

| CPU | Intel Core i5-8600 CPU |
| --- | --- |
| RAM | 16GB |
| GPU | Nvidia TITAN Xp Graphics Card (12GB) |
| Operating System | Ubuntu 18.04 |
| Deeplearing Toolkit | PyTorch 1.7.0 |
| Detection Codebase | mmdetection 2.6.0 |

**Supplementary Table 6:** Key Hyperparameters for Training leukocyte Detectors

| Optimizer | Stochastic Gradient Descent, |
| --- | --- |
| Learning Rate | 0.01 |
| Momentum | 0.9 |
| Weight Decay Coefficient | 0.0001 |
| Number of Epoch | 16 |
| Batch Size | 16 |
